# Plasmonic-Ceria Nanoparticles as Fluorescence Intensity and Lifetime Quenching Optical Sensor

**DOI:** 10.3390/s18092818

**Published:** 2018-08-27

**Authors:** Nader Shehata, Effat Samir, Ishac Kandas

**Affiliations:** 1 Center of Smart Nanotechnology and Photonics (CSNP), SmartCI Research Center, Alexandria University, Alexandria 21544, Egypt; effat_samir@mena.vt.edu (E.S.); ishac@vt.edu (I.K.); 2 Department of Engineering Mathematics and Physics, Faculty of Engineering, Alexandria University, Alexandria 21544, Egypt; 3 USTAR Bio-Innovation Center, Utah State University, Logan, UT 84341, USA; 4 Kuwait College of Science and Technology, Doha Area, 7th Ring Road, Safat 13133, Kuwait; 5 The Bradley Department of Electrical and Computer Engineering, Virginia Tech, Blacksburg, VA 24061, USA; 6 Department of Electrical Engineering, Faculty of Engineering, Alexandria University, Alexandria 21544, Egypt

**Keywords:** plasmonic, gold nanoparticles, ceria, fluorescence, sensor

## Abstract

Ceria nanoparticles have been recently used as an optical fluorescent material with visible emission under ultraviolet excitation, due to the formation of trivalent cerium ions with corresponding oxygen vacancies. This paper introduces the enhancement of both fluorescence emission and lifetime through adding gold nanoparticles. The reason is due to possible coupling between the plasmonic resonance of gold nanoparticles and the fluorescence emission of ceria that has been achieved, along with enhanced formation of trivalent cerium ions. Both factors lead to higher fluorescence intensity peaks and shorter fluorescence lifetimes. As an application, gold-ceria nanoparticles have been used as an optical sensing material for lead particles in aqueous media based on fluorescence quenching. Stern-Volmer constant of in-situ gold-ceria nanoparticles is found to be 2.424 M^−1^, with a relative intensity change of up to 40% at 0.2 g/L.

## 1. Introduction

There are different fluorescent nanostructures which can be used as optical nanosensors based on fluorescence quenching mechanisms. One of the most efficient fluorescent nanostructures are cerium oxide (ceria) nanoparticles, based on their visible fluorescence emission under ultraviolet excitation in addition to their reduction-oxidation (redox) properties [1,2]. Ceria nanostructures can include oxygen vacancies which can be adsorption centers for some quenchers such as tiny metallic particles. Therefore, sensing of quenchers can lead to the variation of both fluorescence intensity and lifetime, which can be used in a wide variety of applications, including biomedical treatment and environmental monitoring [3,4]. The adsorbing centers inside ceria nanoparticles are found according to oxygen vacancies (O-vacancies) associated to the formed trivalent cerium ions inside the crystalline structures of non-stoichiometric ceria CeO_2−x_. The trivalent trap state of cerium ions is correlated to fluorescent visible emission under ultraviolet emission related to 5d–4f energy level transitions [5,6,7]. Regarding the fluorescence lifetime, it measures the time of the fluorophore, which here is trivalent cerium ion, in the excitation state before returning back to the ground state [8]. Hence, fluorescence lifetime is another property affected by various parameters within ceria nanoparticles through different quenchers which must be sensed [9,10]. In another point, adding some plasmonic nanostructures within ceria such as gold nanoparticles can enhance the fluorescence emission due to the coupling between the fluorescence emission of ceria and the plasmonic resonance of plasmonic nanostructures given the overlap of both spectra. In addition, the added gold nanoparticles can lower the activation energy of O-vacancies inside the crystalline structure of ceria, which can lead to more trap states of trivalent cerium ions which are responsible for more fluorescence emission. Therefore, the idea of this paper is to enhance the fluorescence emission by adding plasmonic gold nanoparticles in ceria nanoparticles solution whether in-situ or post-synthesis. Then, both fluorescence intensity and lifetime will be studied for ceria nanoparticles with and without added gold nanoparticles. Then, as an application, our plasmonic-enhanced-ceria nanoparticles have been investigated as optical sensors for detecting tiny metallic particles such as lead (Pb) due to its toxicity and the urgent need to detect it in a wide range of applications [11]. The main idea is related to optical quenching of both fluorescence emission intensity and lifetime due to the static adsorption of the quenchers by the formed oxygen vacancies inside the ceria nanoparticles. Ceria nanoparticles will be synthesized using chemical precipitation technique for its easier preparation procedure and cheap precursors. Ready-made gold nanoparticles are added in both cases; in-situ during the initial precursors additions process, and post-synthesis after the full preparation of ceria nanoparticles. Then, the synthesized plasmonic-ceria nanoparticles have been characterized to measure their optical absorbance, direct allowed bandgap, fluorescence emission intensity, and fluorescence emission lifetime. Then, we can decide which procedure of adding gold gives a better fluorescence emission, whether in-situ or post-treatment. As an application, our synthesized plasmonic-ceria nanoparticles are used as sensing material for tiny metallic particles through the quenching of fluorescence emission intensity and the change in fluorescence lifetime. Then, Stern-Volmer constants have been calculated which gives evidence to the sensitivity of the plasmonic-ceria nanoparticles to optically detect the added quenchers [4].

## 2. Materials and Methods

### 2.1. Materials Synthesis

The cerium oxide nanoparticles synthesis was performed using a chemical precipitation technique for simplicity [12]. For each sample, cerium chloride (Sigma Aldrich, St. Louis, MO, USA, 0.5 g) has been added to distilled water (40 mL) with ammonium hydroxide (1.6 mL) added as a catalyst. Then, the solution is stirred at temperature of 50 °C for two hours at a stirring speed of 500 rpm, then the stirring is continued for 24 h at room temperature. The first heating step is important to avoid the formation of cerium hydroxide. After the full period of stirring, the sample is centrifuged and washed with ethanol to remove any reacted cerium chloride. Gold nanoparticles (Sigma Aldrich) of 20 nm diameter have been selected so that optical plasmonic resonance is in the same range of fluorescence emission of ceria nanoparticles, as will be shown later in the Results section. Gold nanoparticles are added in two ways: firstly added with the initial precursors before the stirring which we call it in-situ, and the second way is to add gold nanoparticles as a post-synthesis step after the full synthesis of ceria nanoparticles. In both modes of gold addition, a 1 mL solution of gold nanoparticles has been added to the ceria solution. Thus, the technique which leads to the highest intensity of fluorescence emission could be checked.

### 2.2. Materials Characterization

Ceria nanoparticles have been characterized with and without added gold nanoparticles. The absorbance, which is helpful in direct bandgap calculations, is measured using a dual-beam UV-Vis-NIR spectrometer (PG 92+, Lutterworth, England, United Kingdom) over a wavelength range from 300 nm to 700 nm. The synthesized gold-ceria nanoparticles are then characterized through a hand-made fluorescence spectroscopy setup. The experimental apparatus consists of a fluorescence quartz cuvette, which contains the solution of ceria-gold nanaoparticles, is exposed to violet excitation from a light emitting diode (LED, Thorlab, Newton, NJ, USA) centered at 430 nm. Then, the fluorescent emission from the synthesized nanoparticles is partially collected through a Cornerstone 130 monochromator (Newport, Irvine, CA, United States). The monochromator inlet is perpendicular to the excitation source for minimum scattering and scanned over the spectrum region of 480 nm to 700 nm. Then, the scanned emission from the monochromator is amplified by an Oriel photomultiplier tube (PMT Newport 77360, Irvine, CA, United States). The amplified emission intensity is detected by a Newport Power meter 1918R. Regarding the fluorescence lifetime measurement, the fluorescence intensity setup is modified to add an optical chopper (MC2000B, Thorlab, Newton, NJ, USA) with a chopping frequency up to 10 kHz, following by the LED excitation source. However, the detection part of the fluorescence intensity is exactly used in measuring lifetime, with total integration time of the PMT is 22 ns, which is much smaller compared to the expected milliseconds lifetime region. For optical detecting of tiny particles, different concentrations of lead (Pb) have been added in the solution of the synthesized nanoparticles. Hence, the quenching of both fluorescence intensity and lifetime is measured at different concentrations of metallic tiny particles. The synthesized nanoparticles are then imaged using a TEM (JEOL, Peabody, MA, United States) with an accelerating potential of 80 KV, with calculating the mean diameter using ImageJ software. XRD analysis of the synthesized nanoparticles is measured using a Rigaku x-ray diffraction via Cu Kα radiation (λ = +0.145 nm).

## 3. Results and Discussion

### 3.1. Materials Characterization

**Figure 1:**
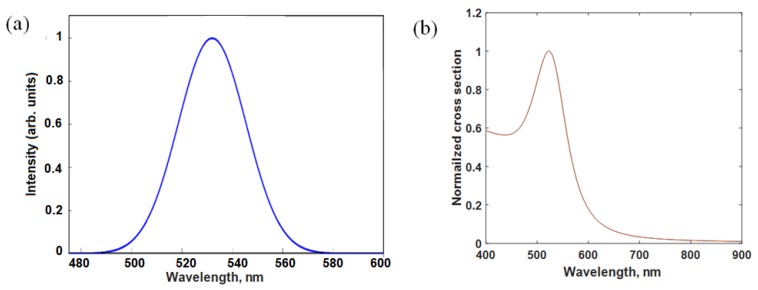
(**a**) Fluorescence emission intensity of ceria nanoparticles with no added gold; and (**b**) absorbption spectrum of gold nanoparticles showing the plasmonic resonance wavelength around 520 nm.

**Figure 2:**
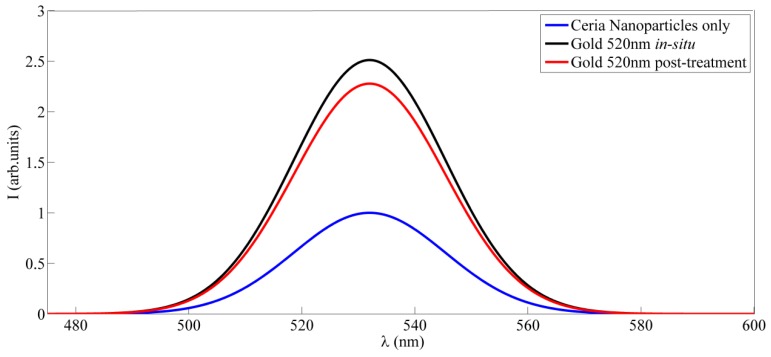
Fluorescence emission of gold-ceria nanoparticles in both cases of in-situ and post-synthesis addition.

**Figure 3:**
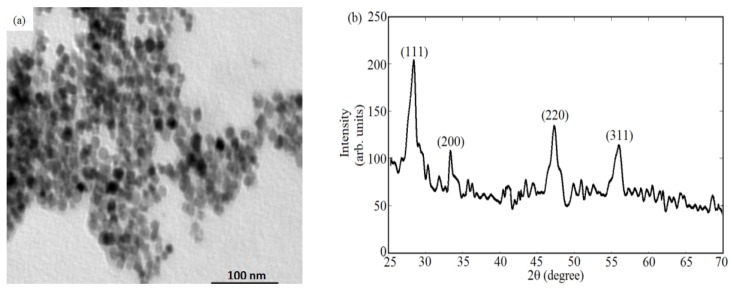
(**a**) TEM image of in-situ added gold-ceria nanoparticles; and (**b**) XRD pattern of gold-ceria nanoparticles.

Figure 1a shows the fluorescence emission of ceria nanoparticles alone without adding gold nanoparticles, under ultraviolet excitation of 430 nm. The added plasmonic gold nanoparticles have the absorption spectrum as shown in Figure 1b. It shows that the plasmonic resonance in the range close to 520 nm which a good match with the fluorescence emission of ceria nanoparticles. Figure 2 shows the fluorescence emission spectrum of ceria nanoparticles with added gold nanoparticles whether in-situ or post-synthesis. Compared to ceria nanoparticles, the in-situ added gold nanoparticles show enhanced fluorescence emission peaks, which is found to be higher than the case of gold added post-synthesis. That proves the possible coupling between plasmonic resonance wavelength of gold nanoparticles with the fluorescence emission of ceria nanoparticles. Figure 3a shows TEM image of in-situ gold-ceria nanoparticles with mean diameter of ceria nanoparticles around 10 nm along with gold nanoparticles of 20 nm diameter. Figure 3b shows XRD pattern of gold-ceria nanoparticles shows the peaks of cerium oxide with no peaks indicating the formation of gold oxide. From the first stable peak of plane (111), ceria nanoparticles mean diameter can be re-calculated using Scherer’s formula [13], and found to be ~10 nm which is compatible with what we obtained from TEM image.

**Figure 4:**
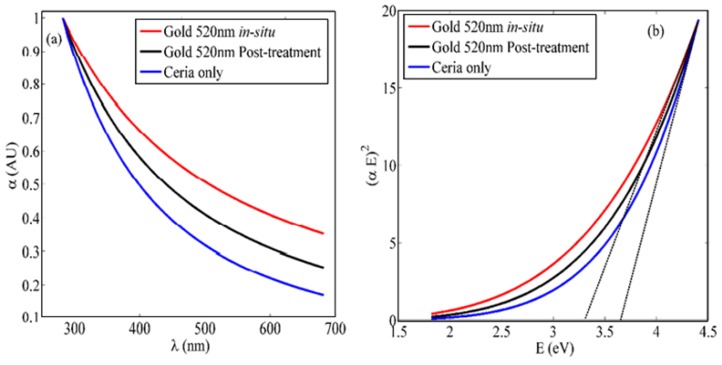
(**a**) Absorbance spectra; and (**b**) bandgap calculations of ceria nanoparticles with and without added gold nanoparticles. The added gold is in both cases of in-situ and post-synthesis. The dotted lines in Figure 4 b refer to the extensions of (*αE*)^2^ curves, which lead to bandgap values.

In addition to the coupling between plasmonic resonance and fluorescence emission, the added gold nanoparticles can help in better formation of trivalent cerium ions. The formation of Ce^3+^ ionization states correspond to a better probability of 5d–4f transition and consequently the enhancement of fluorescence emission intensity. Figure 4a shows the absorbance dispersion of ceria nanoparticles, with and without added gold nanoparticles, along with the corresponding bandgap calculations in Figure 4b. Equation (1) describes how to calculate the allowed direct bandgap from the linear region of the absorbance dispersion curves [14]:

∝*E* = *A*(*E* − *E*_*g*_)^1/2^ (1)

where α is the absorbance coefficient, *A* is a constant that depends on the effective masses of electrons and holes in the material, *E* is the absorbed photon energy, and *E_g_* is the allowed direct bandgap. It can be observed that the in-situ added gold-ceria nanoparticles has lower bandgap closer to 3 eV, compared to the post-treatment case or no added gold. That gives an evidence of the more +3 ionization states of cerium ions, associated with formation of oxygen vacancy, due to added gold nanoparticles. That leads to shift the bandgap of non-stoichiometric CeO_2−x_to 3 eV bandgap range. The formation of more trivalent ions associated with more O-vacancies and higher fluorescence intensity emission, is better in the case of in-situ addition more that the post-treatment case. That can be another possible factor for enhancing the fluorescence emission, in addition to the possible coupling between ceria fluorescence emission and plasmonic waves of gold nanoparticles. Both factors lead to higher fluorescence intensity peaks.

**Figure 5:**
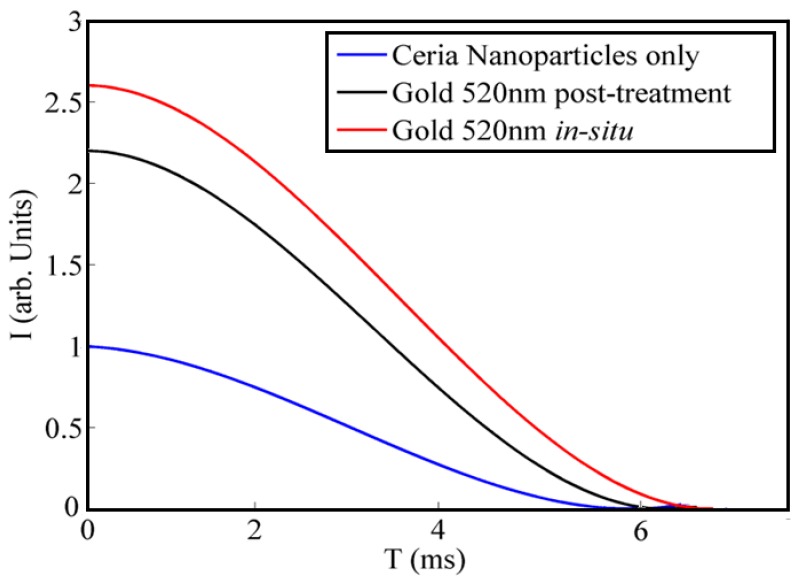
Fluorescence lifetime of ceria and gold-ceria nanoparticles in both cases of in-situ and post-synthesis addition.

Another optical characteristic is shown in Figure 5 related to the fluorescence lifetime measurement of ceria nanoparticles with and without added gold nanoparticles. It can be observed that adding the plasmonic nanostructure reduced the emission lifetime from 4.6 ms in case of no added gold up to 3.9 ms in case of in-situ added gold nanoparticles. The coupling between the plasmonic resonance and fluorescence visible emission under ultraviolet excitation leads to smaller fluorescence lifetime corresponding to higher emission intensity.

### 3.2. Fluorescence Intensity and Lifetime Quenching

**Figure 6:**
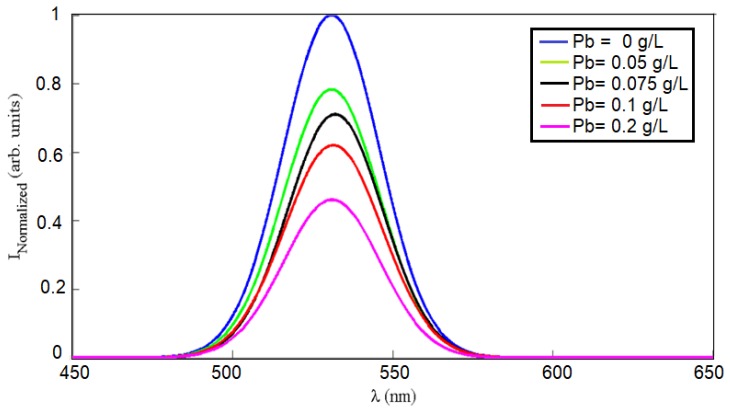
Fluorescene intensity quenching of in-situ gold-ceria nanoparticles at different Pb concentrations.

**Figure 7:**
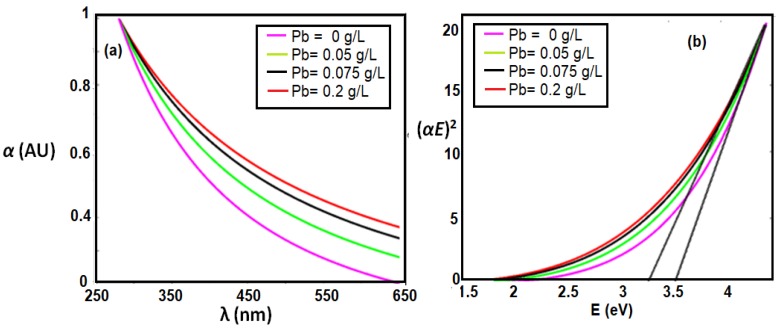
(**a**) Absorbance dispersion; and (**b**) bandgap calculations for gold-ceria nanoparticles at added Pb concentrations to prove the static mechanism of the sensing.

As previously proved, in-situ gold-added-ceria nanoparticles showed the highest fluorescence emission intensity compared to post-synthesis gold-ceria and pure ceria nanoparticles. Therefore, we focus in this section on the application of in-situ gold-ceria nanoparticles as the main sensitive host for tiny particles to be optically detected. Figure 6 shows the fluorescence intensity quenching of gold-ceria nanoparticles with added increased concentration of lead tiny particles. It can be noticed that the fluorescence intensity was reduced up to 40% of its initial emission intensity with added concentration of lead of 0.2 g/L. To prove the mechanism of the quenching whether it is static or dynamic, the absorbance dispersion spectra of gold-ceria nanoparticles are measured with added variable lead concentrations, along with their corresponding bandgap calculations as shown in Figure 7. Both absorbance and bandgap show red shift according to the increased concentration of added metallic particles. That can be explained due to the static adsorption of the tiny particles with the help of the charged oxygen vacancies associated with the more formed trivalent cerium ions, which have been already increased due to the added in-situ gold nanoparticles. Hence, the quenching mechanism here is dominantly static.

**Figure 8:**
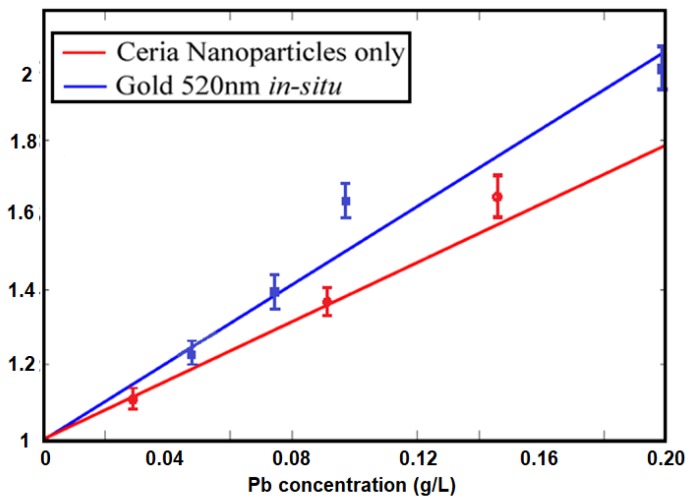
Stern-Volmer calculations of relative quenched fluorescence intensity at different Pb concentrations hosted by the sensing material of gold-ceria nanoparticles.

Based on the intensity change as shown in Figure 6, the relative change in the fluorescence emission intensity at different Pb concentrations is described in Figure 8. The linear relation is analyzed by the Stern-Volmer equation [15]:

*I*_*o*_/*I* = 1 + *K*_*SV*_[*Q*] (2)

where *I*_*o*_ and *I* represent the peak intensities of the steady-state fluorescence in the absence and presence, respectively, of the lead particles quenchers, *K_SV_* is the Stern–Volmer quenching constant which is an indication for the sensitivity of the nanostructure to optically detect the quencher [16], and [*Q*] is quencher concentration; which here is the lead particles. Figure 8 shows relatively linear behavior between the relative intensity and the quencher concentration. The value of the calculated *K_SV_* is 2.424 M^−1^ for in-situ gold-ceria nanoparticles according to the lead quencher, compared to 1.645 M^−1^ for ceria nanoparticles only. That proves the enhancement of sensitivity by around 50% due to the coupling with plasmonic gold nanoparticles.

**Figure 9:**
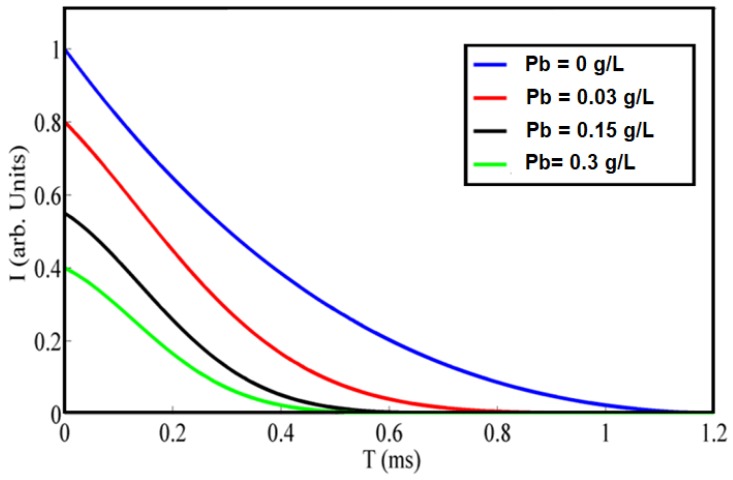
Fluorescene lifetime change of gold-ceria nanoparticles due to different concentrations of lead quencher.

Figure 9 shows the quenched fluorescence lifetime change according to the sensed tiny particles. It is obvious that the fluorescence lifetime has been decreased with added lead tiny particles due to the static quenching of gold-ceria nanoparticles due to the lead quencher. In concentrations up to 0.3 g/L, the lifetime is decreased up to 65% of initial lifetime.

## 4. Conclusions

In this work, the fluorescence emission of ceria nanoparticles has been improved by adding in-situ gold nanoparticles. Ceria nanoparticles with visible emission under ultraviolet excitation have been doped with gold nanoparticles which have a plasmonic resonance wavelength close to the peak fluorescence emission of ceria. The impact of adding gold nanoparticles, whether in-situ or post-synthesis, are found to increase fluorescence intensity and decrease fluorescence lifetime compared to ceria nanoparticles only. Gold nanoparticles contribute in improving the formation of trivalent cerium ions with associated O-vacancies, in addition to the coupling between plasmonic resonance of gold and fluorescence emission of ceria. Based on the fluorescence enhancement, ceria-gold nanoparticles are used as optical sensor for lead tiny particles in aqueous media through fluorescence quenching mechanism with Stern-Volmer constant of 2.424 M^−1^. The quenching mechanism is found to be static based on absorbance dispersion measurements.
